# Application of X-ray Computed Tomography to Verify Bond Failures Mechanism of Fiber-Reinforced Fine-Grain Concrete

**DOI:** 10.3390/ma15062193

**Published:** 2022-03-16

**Authors:** Grzegorz Piotr Kaczmarczyk, Roman Kinasz, Vitaliy Bilozir, Ivanna Bidenko

**Affiliations:** 1 Department of Geomechanics, Civil Engineering and Geotechnics, The Faculty of Civil Engineering and Resource Management, AGH University of Science and Technology, Al. Mickiewicza 30, 30-059 Cracow, Poland; rkinash@agh.edu.pl; 2 Department of Building Structures, Lviv National Agrarian University, V. Velikogo 1, 80381 Dublyany, Ukraine; bilozir.vitaly@ukr.net (V.B.); ivannabidenko1@gmail.com (I.B.)

**Keywords:** fine-grain concrete, bond, industrial computed tomography, numerical simulation, xCT, fiber

## Abstract

This paper proposes the use of X-ray computed tomography (µCT, xCT) measurements together with finite element method (FEM) numerical modelling to assess bond failures mechanism of fiber-reinforced fine-grain concrete. Fiber-reinforced concrete is becoming popular for application in civil engineering structures. A dynamically developing topic related to concretes is the determination of bond characteristics. Nowadays, modern technologies allow inspecting the inside of the element without the need to damage its structure. This paper discusses the application of computed tomography in order to identify damage occurring in the structure of fiber-reinforced fine-grain concrete during bond failure tests. The publication is part of a larger study to determine the bonding properties of Ukrainian steel fibers in fine-grain concrete. The authors focused on the visual evaluation of sections obtained from tomographic data. Separately, the results of volumetric analysis were presented to quantitatively assess the changes occurring in the matrix structure. Finite element analysis is an addition to the substantive part and allows us to compare real damage areas with theoretical stress concentration areas. The result of the work is the identification of a path that allows verification of the locations where matrix destruction occurs.

## 1. Introduction

Economic changes resulting from the intensity of mankind’s activities are forcing society to reuse available materials. Recycling has become an everyday habit, and newer and newer recovery technologies try to ensure minimal waste. New trends are forming, such as *urban mining* [1]. Properly implemented, urban mining process allows for effective recycling of valuable raw materials. In the construction industry, the dynamic development of new-generation concretes, so-called *green concretes,* has been observed [2]. This trend adds additional components to concrete mixes such as recycled aggregate, ash, or recycled steel in order to improve the environmental standard while ensuring the assumed physical-strength properties [3,4].

Construction and demolition waste, commonly referred to as CDW, is increasingly being used in the construction industry [5]. Sources also include recycled tire steel fiber (RTSF) in this group. Recycled tire steel wire is used as a fiber in plain concrete and concrete with recycled aggregate in volume proportions of 0.5 and 1% [6]. The introduction of recycled tire waste into the steel mixture resulted in a decrease in compressive strength with an improvement in tensile test results [7]. It is worth noting the additional challenges associated with corrosion susceptibility [8]. Steel-fiber-reinforced concrete (SFRC) is not only about using waste materials. There are functioning production lines of industrial steel fiber (ISF) e.g., Micro- or Hooked-end fiber. There are differences in the obtained mechanical parameters of mixtures reinforced with recycled and dedicated fibers, but the nature of their behavior remains very similar [9]. Research on one group influences the development of the whole RTSF trend. Industrial-fiber-reinforced concrete has been studied reasonably accurately in terms of compression [10], impact resistance [11], and torsion [12]. SFRC can be incorporated into more complex composites using, for example, steel profiles [13]. Studies of compressive fatigue behavior and failure evolution with similar materials use digital image correlates to determine deformation and strain [14]. The growing interest in SFRC has led researchers to investigate the problem of bonding between fiber and concrete matrix. A step in the analysis of the contact between two materials can be a visual assessment of behavior occurring at their interface. Therefore, for obvious reasons, macroscopic inspection is not possible. Modern technology and computed tomography can provide assistance [15]. The authors see a research gap in the use of CT to verify the bond failure mechanism of fiber-reinforced fine-grain concrete.

Computed tomography is typically associated with medical applications. The first CT scanner was developed in 1973 and its authors—Godfrey Newbold Houns-field and Allan MacLeod Cormack—received the Nobel Prize in 1979. Almost half a century of technology development has made modern tomographic examinations highly automated, safer, and faster [16]. Computed tomography allows nondestructive inspection of elements by determining their parameters. Speaking of the tomography itself, it should also be noted that there are many different techniques available, using different signal sources. Some tomographic technologies have a narrow scope of application. Authors distinguish three groups most frequently used in industry:

- Electrical Resistance Tomography, used to determine slurry flow measurements [17].
- Ultrasound tomography, which can be used to visualize the internal behavior of a concrete structure [18,19].
- X-ray microtomography (XCT, µCT, X-ray CT), covering the entire spectrum of materials [20,21]; applications of extended XCT scanning and neutron CT are also known [22].

**Figure 1:**
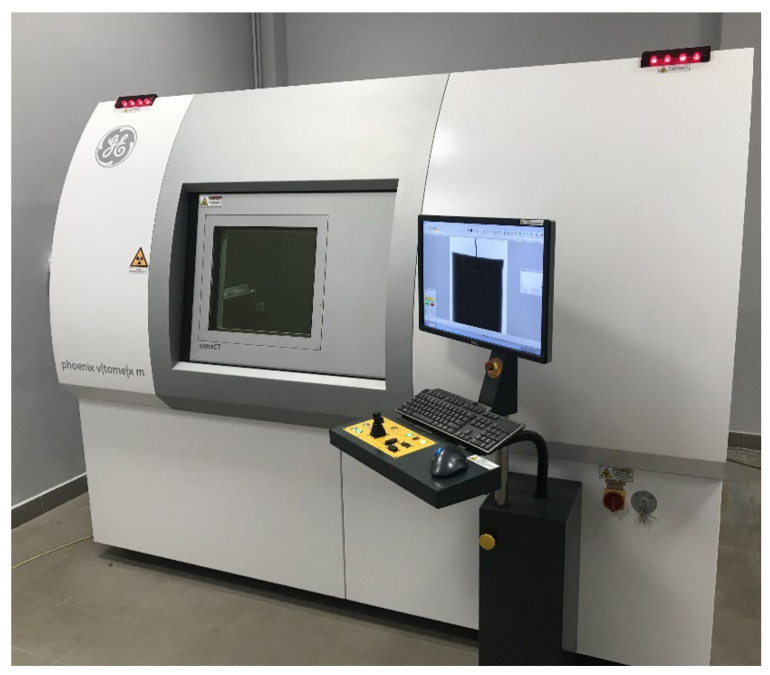
X-ray CT scanner GE phoenix v|tomex|m.

**Figure 2:**
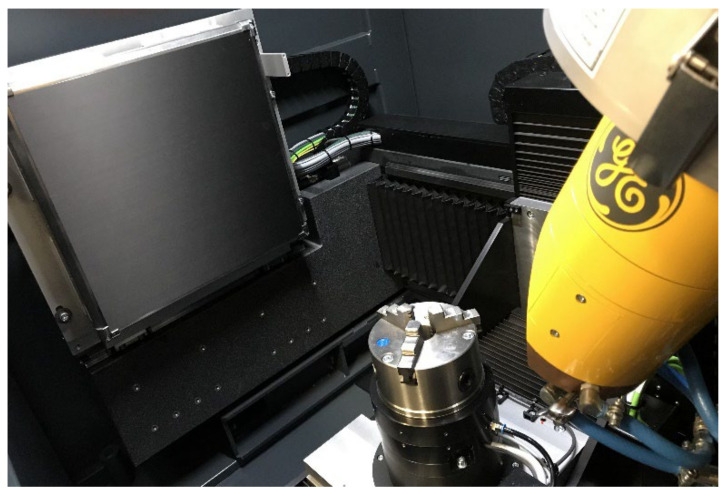
The tomographic chamber of the cone beam device.

X-ray tomographs used in industry are based on X-ray spectroscopy. A detailed description of the principle of operation was published in [20]. The device has been in use for more than 20 years and the information on its concept of operation has not become outdated. An illustrative device is shown in Figure 1. An object is inspected in a special chamber of the device. The item is placed between the radiation emitter, the so-called lamp, and the detector. The device is controlled by determining the position of the object in the space between the lamp and the detector, entering the value of voltage and current generating radiation (power), and determining the operating characteristics of the detector. Appropriate selection of parameters allows the acquisition of results enabling further 3D reconstruction of the object. In contrast with tomography used in the practice of medicine (where the comfort and health of the patient is a priority), during an industrial examination, the detector and the lamp remain in a fixed position, while the sample is rotated along the vertical axis. In the course of a single scan, thousands of absorption measurements of the radiation beam penetrating the examined object are made. A schematic view of the cone beam tomograph and the inside of the chamber are shown in Figure 2.

The next step is the reconstruction of the 3D volume. During a full rotation, the device generates thousands of images in high grayscale (e.g., the GE Phoenix v|tomex|m uses 14 bit). The most common is the implementation of complex algorithms at the reconstruction stage, resulting in correction of images such as beam hardening correction, automatic geometry calibration, and geometry optimization [23,24]. The reconstructed object can be visually inspected by analyzing 2D cross-sectional images, 3D images, and further volumetric analysis. Modern CT images are characterized by highly detailed detectability, allowing details <1 µm to be seen [25].

The current software offers a number of built-in analysis types that are helpful when working with CT data. The leading analyses are porosity, geometric deviation, and material orientation analysis. The software also allows for FEM calculations and flow simulations. [26]. Sources indicate the use of data obtained from CT scans to evaluate the compactness of concrete [27]. The method was also used to describe the surface of concretes [28]. Existing methods for working with CT-acquired data are described extensively in the publication [27]. Studies on the effect of static loading on concrete specimens using tomographic imaging have been reported in [29]. The usefulness of the tomographic image method in evaluating shear tests is pointed out in [30]. There are studies that show how to determine the change in pore size of a concrete sample undergoing freezing cycles by tomography using a medical device [31]. Basic tomographic studies of steel-fiber-reinforced concretes are presented in [32]. The tomographic analysis mainly focused on the orientation of fibers in space and the cracks formed during the strength tests.

An extensive review of the analysis of concrete and asphalt construction materials is presented in [33]. According to [34], porosity analysis of concrete materials by microCT is popular because of the inherent porosity of such materials and the important role that pores can play in mechanical and transport properties. There are significant correlations between pore size distribution and concrete strength [35], and the transport properties of concrete [36]. Another application that researchers find for computed tomography in the analysis of fiber-reinforced concrete is the analysis of fiber distribution. A homogeneous distribution is a key factor for the correct behavior of a fiber concrete element. Fiber orientation has been clearly indicated by CT in [37]. At the same time, in [38], computed tomography was used to determine fiber distribution in macro-plastic fiber-reinforced concrete slab-panels, indicating the versatility and usefulness of CT in analyzing the distribution of fibers of different densities.

## 2. Materials and Methods

### 2.1. Specimens and Strength Testing

**Figure 3:**
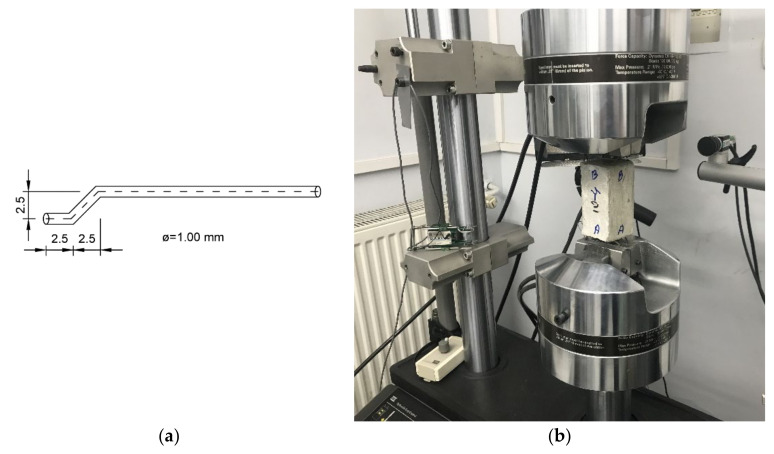
Laboratory testing of concrete specimens with fibers: (**a**) nominal fiber geometry; (**b**) MTS measurement system with test specimen.

The essence of the planned strength tests was to determine the forces required to pull out the fibers of the concrete cubes. Hooked-end fibers were placed in the concrete cubes (Lviv National Agrarian University, Lviv, Ukraine. Prisms 50 × 50 × 100 mm were made of fine-grained concrete. To vary the results, the fibers were anchored at three lengths: 10, 15, and 25 mm. The geometry of the fiber is shown in Figure 3a. The bend results in improved bonding, and fibers with this shape are widely used. The tested samples were based on concrete with the following formulation: cement M400 444 kg/m^3^, water 240 l/m^3^, sand 1644.43 kg/m^3^. The w/c ratio was kept at 0.54.

In order to obtain general concrete parameters, 12 cubic concrete samples were subjected to uniaxial compression testing according to the procedure in [39]. The test series were tagged as Series 10, Series 15, and Series 25, respectively. Strength corresponds to the concrete class C25/30. This study is a continuation of the research conducted by the authors’ international group [40,41]. During the test, one end of the specimen was fixed. The other end was acted upon by force. The detailed methodology is described in [40]. An analytical approach for determining bond parameters is presented in [41]. Testing was performed on the machine shown in Figure 3b. During the test, displacement was controlled. The pullout speed was set at 0.05 mm/s. The adopted methodology is in accordance with the review in [42].

A similar study, but on materials of a different type, was presented in [43], where the essence of the study was to determine the chemical bond properties of Polyvinyl Alcohol—Engineered Cementations Composite. The authors took a similar approach to strength testing.

### 2.2. CT Scan

The tomographic examinations were performed on a GE Phoenix v|tomex|m device (General Electric Company, Hürth, Germany) with a microfocus lamp using a cone beam. The warm-up and centering procedure was carried out on the instrument. In order to test irregularly shaped specimens, the samples were mounted on a low-absorbent foam for stable mounting on a manipulation table. The specimen was positioned to focus the viewing range on the fiber/concrete interface—the Region of Interest (ROI) option was selected. The ROI focuses the scope of the test on a portion of the specimen allowing us to select the section of interest. Each specimen was scanned twice, before and after the strength test, with the same scanning parameters—130 kV at 250 µA. A copper filter of constant thickness was used during the test. The measurement was performed with an accuracy at which the voxel dimension was equivalent to 30 µm. A calibration procedure was performed according to the manufacturer’s recommendations. One examination assumed the taking of 2700 images. A single run lasted approximately 50 min. During the examination, the detector’s shift module was activated to exclude the effect of defect of a single detector pixel and the auto-sco function, which allows for geometry optimization. For safety reasons, the radiation level was monitored during and after the examination.

### 2.3. Volumetric Analysis

The first task was to reconstruct separate 3D solids based on data from separate scans. The program provided by the manufacturer of the tomograph (General Electric Company, Hürth, Germany), datos|x, was used for this purpose. During the reconstruction, the beam hardening correction, automatic geometry calibration, and geometry optimization algorithms were used. The reconstructed 3D geometry allows for visual inspection and further analysis.

**Figure 4:**
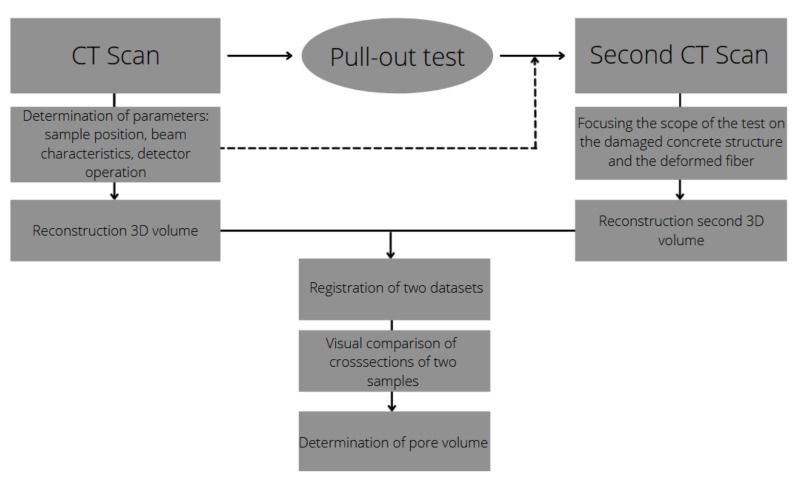
Data workflow diagram.

Afterwards, a registration of two scans (before and after pulling out the metal element) was performed. Scanning a component using ROI leads to one fundamental difficulty: there is no complete image of the outer edges, making it difficult to bring the two scans into a common coordinate system. In order to establish a common position, an internal algorithm was developed. The procedure required determining the porosity of two samples. Then, the coordinates of the 11 largest pores and the coordinates of the centers of gravity of the two samples were entered as input to the script. The transition between coordinate systems was determined. In successive steps, the coordinates of progressively smaller pores were compared. The 3D rotation angles and the displacement vector of one of the samples were output. Fitting was assessed visually. After 10 shift-rotation iterations, a correct match between the two data sets was found. A diagram of data workflow is shown in Figure 4.

Porosity determination is widely used in concrete testing [33,44] and describing changes in the material caused by external factors. Leading CT software (3.4, Volume Graphic, Stockholm, Sweden) allows automated porosity/inclusion analysis. Before the porosity analysis were calculated, the absorption characteristics of three materials—steel, concrete, and air—are defined. The process of determining the volume of air voids was carried out on the reconstructed 3D solid. The VGDefX algorithm set to Voids mode was used. The pores were considered significant if their volume exceeded 8 voxels. The analysis reveals air voids in the entire solid or in a portion of the solid indicated by the operator. The evaluation is performed visually and based on tabular summaries and graphs. Depending on the void size, the pores are visualized with different colors on the cross-sections and in the 3D view. In addition, the distribution of pores as a function of the respective coordinate is clearly measurable.

### 2.4. Numerical Simulations

The development of tomographic analysis allows us not only to evaluate data inside a given CT software, but also to further transform the obtained data and perform strength analyses. The dedicated software has a built-in Finite element method (FEM) calculation module; however, it is only designed for basic simulations [26]. The authors of this paper note the great potential for applications of microtomography-acquired data (especially geometric data) in numerical simulations. So far, sources [45] indicate the use of tomographic images to reconstruct the sample geometry as a mesh grid.

The authors decided to develop models of fibers that could be numerically extracted from a three-dimensional (3D) concrete block. The CT data allow us to reconstruct the geometry in two ways: develop a surface consisting of triangular planes or define the geometry by using simple solids (e.g., cubes, spheres, or cylinders). The authors’ experience shows the wide applicability of the mesh method when working with samples with complex geometries. Fine triangles very accurately described the surface of, for example, a transverse flute, the model of which was used to simulate airflow. The high variability of the geometry justified the use of a complex meshing method, which in further steps, needed powerful computational software and high computing power. The second approach involves replacing the geometry of a given element by fitting basic 3D solids. The solids are matched in a semiautomated way. The approach enables fast and fairly accurate reproduction of the actual geometry of uncomplicated shaped elements such as simple rods, cubes, or rings. The approach allows for optimal use of computing power and a better representation of the geometry than the popular 3D extrude. Due to the variable shape of the fibers, it was deemed necessary to develop an accurate model containing a large number of triangles.

**Figure 5:**
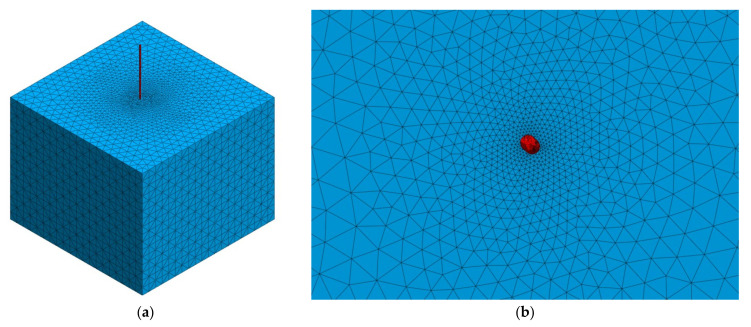
Mesh model: (**a**) isometric view of the mesh; (**b**) top view—finite element size progression.

The purpose of the analysis was to determine the stresses occurring at the contact between the steel fiber and the concrete. The assumed numerical verification would allow us to determine where the failure of the concrete structure may occur. In the course of the work, the focus was on accurate mapping of individual fibers extracted from concrete specimens. The fiber defined by the triangles was subtracted from the concrete cube. Visually, in Figure 5, the fiber is shown in red and the concrete in blue. Due to the innovative approach to the applicability of CT data in the numerical calculations, the distribution of aggregate and air pores in the concrete mix was neglected. When working with this type of data, the process of meshing 3D solids can be problematic. The calculation of a specimen where the fiber has a radius of 0.5 mm required the use of extremely small finite elements. A manual check of the mesh size was made and it was determined that the fiber finite elements should be 0.15 mm in size. Optimization of the mesh size allowed for variation in the size of elements responsible for the concrete structure—denser elements in the immediate area of the fiber resulted in higher accuracy of the numerical solution, while larger elements (5 mm) at the edges allowed for shorter computation time. The resulting models consisted of approximately 3 million tetrahedral finite elements. The calculations were performed in midas GTS NX software (2019 ver. 1.2, MIDAS Information Technology Co., Seongnam, Republic of Korea).

The boundary conditions assigned to the models were full fixation of the lower nodes of the concrete mesh and static load applied to the upper edge of the fiber. The assigned force was determined according to MTS press pullout tests and was taken as the series average value. The material properties for the concrete and fiber were assigned according to tests that are the basis for another article. The data loaded into the numerical software (2019 ver. 1.2, MIDAS Information Technology Co., Seongnam, Republic of Korea) (Midas GTS NX) are shown in Table 1.

## 3. Results

### 3.1. Strength Testing

Tests were conducted by pulling the fiber out of the concrete. The test was performed on an MTS Model 685.53 machine. The displacement and force values were recorded during the tests. The exact force values are shown in Table 2. The test results and analytical approach are the basis for the analyses that are part of a separate study conducted by the team. Detailed results are described in the publication [40]. Relevant to the subject of the thesis is the fact of the quasi-linear increase in the force required to pull out the fiber. At this stage, the team hypothesized that the main damage to the structure would be expected within the bend of the fiber. The team consider that the structure of the concrete and the porosity of the matrix in the immediate vicinity of the fibers was important during the study.

### 3.2. Computed Tomography

The purpose of the visual inspection was to determine the potential damage occurring in the concrete structure due to the pullout force. By analyzing the scans of the samples before mechanical action with 10 mm anchorage, the contact surface between the fiber and matrix of good quality can be observed. In these specimens, no additional spaces or pores of large volume were observed at the reinforcement–matrix interface. The test did not reveal additional cracks. The aggregate was distributed homogeneously throughout the concrete. Pulling out the metal element caused visible changes within the direct contact area between the two materials. There are visible cracks in the concrete at the edge of the sample. Part of the loose material was removed during the test, reducing the volume of the concrete element. The changes are particularly visible at the top edge of the specimen and at the height of the metal bend. Visually, the changes are interpreted as a result of tensile and shear stresses acting on the matrix.

The reconstructed specimen with an anchorage length of 15 mm revealed a higher overall porosity of the concrete. Visually, pores are assessed that they have a larger diameter. In visual assessment, the difference from the specimen with anchorage length of 10 mm is found to be significant. Air spaces between fiber and concrete are locally observed. No cracks are seen in the concrete structure. Similar to the previous specimen, no aggregate sedimentation occurred. The pullout of the fiber led to the destruction of the concrete in the area where the bending of the steel element occurred. Some of the loose material was removed during the test, reducing the volume of the specimen.

**Figure 6:**
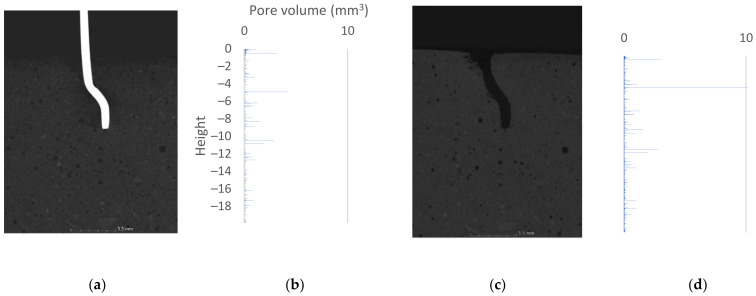
The 10 mm sample: (**a**) 2D cross-section before fiber pullout; (**b**) 3D porosity analysis—plot of pore volume against height in sample before fiber pullout; (**c**) 2D cross-section after fiber pullout; (**d**) 3D porosity analysis—plot of pore volume against height in sample after fiber pullout.

**Figure 7:**
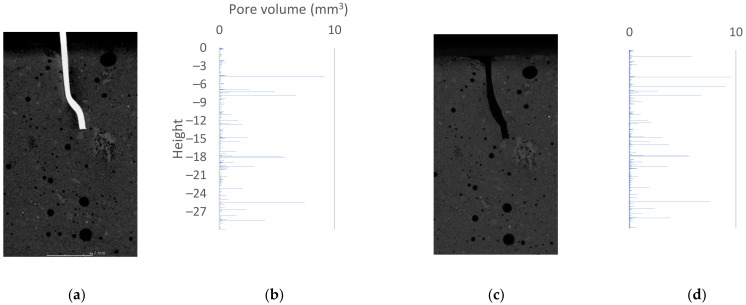
The 15 mm sample: (**a**) 2D cross-section before fiber pullout; (**b**) 3D porosity analysis—plot of pore volume against height in sample before fiber pullout; (**c**) 2D cross-section after fiber pullout; (**d**) 3D porosity analysis—plot of pore volume against height in sample after fiber pullout.

**Figure 8:**
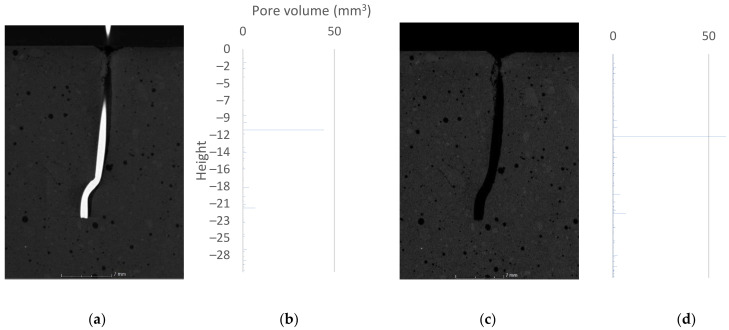
The 25 mm sample: (**a**) 2D cross-section before fiber pullout; (**b**) 3D porosity analysis—plot of pore volume against height in sample before fiber pullout; (**c**) 2D cross-section after fiber pullout; (**d**) 3D porosity analysis—plot of pore volume against height in sample after fiber pullout.

The cross-section of the specimen with an anchorage length of 25 mm indicated a situation similar to the first specimens. Small air pores, uniformly distributed, are visible in the matrix. Locally, the fiber is surrounded by air pores. Cross-section analysis indicated fine cracks in the concrete around the upper contact zone. The pullout test led to crack propagation, but the material was not pulled out together with the steel fiber. The cross-sectional images are shown in Figure 6, Figure 7 and Figure 8a,c.

### 3.3. Volumetric Analysis

The primary tool used to describe the volume changes is porosity analysis. Figure 6, Figure 7 and Figure 8 summarize the 2D cross-sections and 3D porosity analysis results, reduced to pore volume dependence plots shown on the vertical axis. The horizontal bars represent the volume of pores with a geometric center at a given height. The beginning of the coordination system was taken to be the upper surface of the analyzed sample. In Figure 6, Figure 7 and Figure 8, the beginning of the height axis corresponds to the concrete surface. Cylinders of 2.5 cm diameter were analyzed. The heights of the cylinders are 20, 30, 30 mm respectively. Voxels belonging to fibers were excluded from the analysis—the void created by removing the steel element did not affect the results obtained. The following graphs show pore volumes in voxels. A voxel is the basic cell of data acquired during the tomography process. A voxel of a 3D solid can be compared to a pixel in a 2D image. A local increase in porosity indicates that destruction has occurred at a particular location in the structure under analysis.

Figure 6 shows the analysis results of the sample with the fiber being anchored to a depth of 10 mm. Figure 6b,d show the results of porosity analysis before and after the fiber pullout test. Except for isolated differences, the graphs are visually consistent with each other, which speak to the correctness of the applied methodology. The main changes can be seen in two places on the graph: at 0.49 mm and at 5 mm depth. The analysis of visualization of the data indicated that the change in porosity in the range of 0–1 mm was a result of propagation of cracks created during the process of pulling out the fiber.

At a depth of 5 mm, the most significant change in structure occurred. The pore volume changed from 4 mm^3^ to over 10 mm^3^. Thus, there was more than a doubling of the local air space in the bend zone of the tested fiber. The increased pore volume is due to concrete deterioration and pullout of the loose material. The destruction occurred at the locations where the air pores between the fiber and concrete were noted.

When analyzing the sample with an anchorage length of 15 mm (Figure 7), additional pore volumes are noticeable, appearing after the fiber pullout test. The largest changes are seen at 1.5 and 6.5 mm. The results indicate destruction of the concrete structure within the upper contact zone—the zone near the surface of the sample. Large destruction can be seen on the lower side of the fiber curve (depth of approximately 6 mm). Destruction occurred on a smooth surface. Pulling out the fiber resulted in widening of the hole. In Figure 7c, an additional air pocket caused by fiber extraction is clearly visible.

The results for the sample with an anchorage length of 25 mm (Figure 8) indicate the presence of an air void between the fiber and the concrete. Its geometric center is located at 11 mm length. Local air voids, about which the authors wrote in the previous subsection, create a single, connected space, which locally, on individual cross sections, appears to be a separate air pocket. Based on the analysis of CT data, it can be clearly stated that adhesion forces were not transferred through the entire fiber surface. Pulling out the fiber resulted in ejection of the damaged and loose material. It should be noted that all specimens with an anchorage length of 25 mm had air voids in the “before fiber pullout test” condition. Damage to the matrix is visually apparent from the surface to a depth of 2 mm. Microcracks smaller than 2 voxels (60 µm) were not captured by the VGDefX algorithm, which was focused on finding pores larger than 8 voxels.

### 3.4. Numerical Simulations

**Figure 9:**
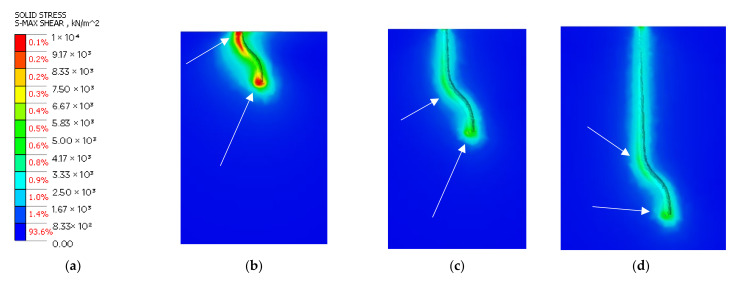
Cross-sectional results of 3D FEA analysis: (**a**) legend; (**b**) 2D cross-section of the model anchored at 10 mm-depth; (**c**) 2D cross-section of the model anchored at 15 mm-depth; (**d**) 2D cross-section of the model anchored at 25 mm-depth.

The variable geometry of the fibers intuitively suggests that the stresses from the pullout test will not be consistent along the length of the element. The advantage of numerical methods over analytical approaches is the ability to input a geometrically and materially complex system. The models developed on the basis of tomographic investigations, assuming appropriate materials, closely represent the real stress concentrations in the tested specimens. The results are summarized in Figure 9. Stress maps show shear stresses from the contact between fibers and fine-grain concrete. The visuals have been scaled so that the color range is consistent in all three images. Stress concentration locations were indicated graphically by white arrows.

The highest shear stress values were obtained for the model anchored at 10 mm (Figure 9b). For this specimen, the highest strain is observed at both edges of the contact surface and reaches a value of about 10 MPa. The increased stresses occur locally. Increasing the anchorage length to 15 mm caused a decrease in the values of local stress concentrations. As in the previous model, the concentrations occur at the lower end of fiber and at the location of the element course change. The stresses exceed the value of 4 MPa. For the specimen with 20 mm anchorage, the increased stress level is noticed under the lower end of fibers. The maximum stresses reach the value of 4.07 MPa. In addition, the increased stress level occurred at the location of the change in the course of the element.

The last test containing specimens anchored at a depth of 25 mm globally indicated another reduction in stresses. As in the previous cases, the stress concentrations occur at the bottom part of the course change and just below the top surface. By identifying low stresses, the FEM analysis indicates that potential failure may occur in areas where stresses are induced by random factors such as porosity or material weakening related to the setting of the concrete itself.

The shear stresses are found locally just below the surface of the concrete and are characteristic of all three experimental models. The authors identify the lower left side of the bend as the second such location (assuming a consensus datum with that shown in Figure 9). The third location for the transfer of forces from the fiber to the dies is the bottom edge of the metal member. The last location is the result of internal forces and horizontal displacements of the end due to deformations of higher elements. The shear stresses are concentrated at the contact surface between the materials. No shear surface formation is observed inside the matrix. The numerical experiment indicates a failure model in which no damage occurs in the deep concrete structure due to adhesion, as proven by the low range of stress concentration. The bond strength is exceeded by generating excessive forces on the fiber flanks. However, damage to the concrete can appear locally.

## 4. Discussion

In the course of the work, the locations where deterioration of the concrete structure occurs were identified. The data were processed visually and by porosity analysis. The bar graphs in Figure 6, Figure 7 and Figure 8 clearly show where the damage occurs. When comparing the tomographic data with the results of the 3D FEM analysis, similarity is noted in the locations of increased stresses and increased pore volume. The authors believe that the movement of fibers itself is a key factor in the experiment. Once the bond failure occurs, pullout can appear in the following cases: when there is damage in the matrix structure (especially in the area of the component bends) or when there are plastic changes in the shape of the metal component. The authors claim that in the analyzed case, an intermediate situation occurs, where locally acting stresses and their extents indicate partial destruction of the matrix (pullout of the destroyed material) and partial plastic deformation of fibers. Visual view of the destroyed element unambiguously indicates the fact of its shape change. The authors are in agreement with the pullout behavior of hooked-end fibers described in [42]. The authors acknowledge that the fiber must undergo plastic deformation during the test. The theoretical behavior presented in the publication [42] should be expanded to include possible matrix damage. An additional aspect not addressed in the source is the effect of pores on the bonding phenomenon. Considering the fiber–pore size ratio, the statistical distribution of porosity in the matrix should be taken into account.

The FEM analysis did not include porosity information. During the visual evaluation of the tomographic data, attention was given to the concentration of damage around the existing pores. As can be seen in [46], air pores also exist between the reinforcing bars and the concrete structure, which has little effect on the bonding phenomenon that is the essence of reinforced concrete construction. The distribution, size, and shape of the pores directly affect the safety of the structure. In their work with fiber reinforced concrete, the authors have identified pores as critical locations where the edges of the concrete structure fail. Again, the authors of this paper would like to focus on the size ratios of pores and fibers, and pores and reinforcing bars. Taking into account the small size of the fiber element, pores are described as an important factor influencing the bonding phenomenon.

Regarding the material itself, the authors see practical applications for Steel-Fiber-Reinforced Concrete. The results of the experimental study on bended beams with dimensions 1500 × 150 × 60 mm [47] seem to be worth quoting. The program required three beams with the same geometry: a nominal beam, a beam with added fibers in the amount of 1.59 kg, and beam with 2.12 kg of fibers, which correspond to the coefficient of fiber reinforcement in volume, ρ_fv_ = 1.5% i 2%, respectively. The beams were loaded with concentrated force in the middle of the span. The experimentally obtained bearing capacities were 6082.5, 7055.5, and 7351.1 N∙m, respectively. The use of fiber reinforcement not only improved the strength parameters but also reduced the crack propagation, which indicates the effectiveness of the SFRC trend.

The authors of article [48] presented the next step in the study of SFRC materials. They described the results of pullout tests on rebar while the concrete was additionally reinforced with steel fibers. From the failure mode studies, it was observed that the addition of 1% hooked-end steel fibers can change the failure mode of pullout specimens from premature splitting to pullout failure. The main role of the steel fibers is not necessarily to increase the strength of the bond; instead, the main role of steel fibers is to increase the ductility of the concrete, which is necessary for high-strength concrete that is prone to brittleness.

## 5. Conclusions

In this paper, the authors attempted to present the possibilities of tomographic image analysis. Modern approaches to research require the knowledge of modern instruments from researchers, both in terms of measurements and for further analysis. Current technology of tomographic imaging and dedicated software allows for complex defect analysis. The research itself requires experience of the operator and the ability to select parameters in such a way that the reconstructed solid is a set of high-quality data. The research can be summarized by the following conclusions:

(1) Data processing should be carried out in two ways, and each analysis requires a visual assessment. When using the results of the analysis, it is necessary to remember the assumptions made (e.g., as to the range of size and shape of the pore in the porosity analysis), which is verified visually. Obtained images present critical areas from the point of view of bond. Industrial computed tomography allows to assess the interior of the material without interfering in its structure. The obtained cross-sectional images make it possible to unequivocally determine the areas where the stresses occurring in the concrete exceeded its strength and changes in the material occurred.
(2) Numerical models can be developed from CT data. The authors processed the data to generate models on which finite element simulations were performed. The simulation results identified the stress concentration locations. Both volumetric porosity analysis and numerical simulation indicated the same locations where damage can occur.
(3) In the course of the work, contact characteristics between materials were derived from the authors’ assumptions. The combination of tomography and strength tests is a complex issue. The study can be deepened by taking into account the randomness of the pore distribution in the matrix.

Based on the tomographic data, it is possible to determine a statistical model of the pore distribution and implement it into the model using, for example, Voronoi tessellation [49], or by reconstructing the geometry directly from an exact sample. Further studies are planned to use the unique Deben CT5000 in situ loadcell tensile stage for X-Ray CT applications. Additional testing would capture changes in the sample structure over the course of the test, which may result in an accurate description of the contact properties between materials. Thus, the next step would be to develop a fully dynamic simulation of the pullout test. Adjusting the dynamic contact parameters of a fiber with a complex geometry will also allow accurate determination of the internal forces.

## Figures and Tables

**Table 1:** Comparison of point cloud mesh to CAD model—cumulated absolute.

Parameter	Fine-Grain Concrete	Steel Fiber
Model Type	Mohr–Coulomb	Elastic
Density (kg/m^3^)	2000	7850
Elastic Modulus (GPa)	31	210
Poisson’s Ratio	0.15	0.30
Frictional angle (deg)	31	Linear elastic material
Cohesion (MPa)	3.86	

**Table 2:** Comparison of point cloud mesh to CAD model—cumulated absolute.

Anchorage (mm)	Pullout Force (kN)	Anchorage (mm)	Pullout Force (kN)	Anchorage (mm)	Pullout Force (kN)
10	218.66	15	309.27	25	250.44
10	184.35	15	231.88	25	357.17
10	121.53	15	179.23	25	308.23
10	264.71	15	208.71	25	316.11
10	281.32	15	261.56	25	271.74
Average	214.11		238.13		300.74

## Data Availability

The data that support the findings of this study are available from the corresponding author upon request.
